# Decreased circulating CXCR3 + CCR9+T helper cells are associated with elevated levels of their ligands CXCL10 and CCL25 in the salivary gland of patients with Sjögren’s syndrome to facilitate their concerted migration

**DOI:** 10.1111/sji.12852

**Published:** 2019-12-13

**Authors:** Sofie L. M. Blokland, Andreas Kislat, Bernhard Homey, Glennda M. Smithson, Aike A. Kruize, Timothy R. D. J. Radstake, Joel A. G. van Roon

**Affiliations:** ^1^ Department of Rheumatology & Clinical Immunology University Medical Center Utrecht Utrecht University Utrecht The Netherlands; ^2^ Department of Immunology Laboratory of Translational Immunology Utrecht University Utrecht The Netherlands; ^3^ Department of Dermatology University of Düsseldorf Düsseldorf Germany; ^4^ Takeda Pharmaceuticals International Cambridge MA USA

**Keywords:** chemokine receptors, chemokines, salivary glands, Sjögren's syndrome, Th cells

## Abstract

CCR9 + T helper (Th) cells can induce Sjögren‐like symptoms in mice and both CCR9 + Th cells and their ligand CCL25 are increased in the salivary glands of primary Sjögren's syndrome (pSS) patients. Increased circulating CCR9 + Th cells are present in pSS patients. CCR9 + Th cells are hyperresponsive to IL‐7, secrete high levels of IFN‐γ, IL‐21, IL‐17 and IL‐4 and potently stimulate B cells in both patients and healthy individuals. Our aim was to study co‐expression of chemokine receptors on CCR9 + Th cells and whether in pSS this might differentially affect CCR9 + Th cell frequencies. Frequencies of circulating CCR9 + and CCR9‐ Th cells co‐expressing CXCR3, CCR4, CCR6 and CCR10 were studied in pSS patients and healthy controls. CCL25, CXCL10, CCL17, CCL20 and CCL27 mRNA and protein expression of salivary gland tissue of pSS and non‐Sjögren's sicca (non‐SS) patients was assessed. Chemotaxis assays were performed to study migration induced by CXCL10 and CCL25. Higher expression of CXCR3, CCR4 and CCR6 but not CCR10 was observed on CCR9 + Th cells as compared to cells lacking CCR9. Decreased frequencies of circulating memory CCR9 + CXCR3+ Th cells were found in pSS patients, which was most pronounced in the effector memory subset. Increased salivary gland CCL25 and CXCL10 expression significantly correlated and both ligands functioned synergistically based on in vitro induced chemotaxis. Decreased memory CXCR3 + CCR9+ Th cells in blood of pSS patients may be due to a concerted action of overexpressed ligands at the site of inflammation in the salivary glands facilitating their preferential migration and positioning in the lymphocytic infiltrates.

## 1 INTRODUCTION

Primary Sjögren's syndrome (pSS) is a chronic autoimmune disorder characterized by oral and ocular dryness and lymphocytic infiltration of the exocrine glands.^1^ A large part of the lymphocytic infiltrates consists of memory CD4 + T cells, which have been shown to play a major role in the immunopathology of Sjögren's syndrome, amongst others by contributing to B cell hyperactivity.^2^ Severe lymphocytic infiltration of minor salivary glands is often accompanied by large numbers of activated B cells, which also play a key role in pSS immunopathology.^3^ Recruitment of leucocytes to tissues is dependent on chemokine‐chemokine receptor interactions and adhesion molecules. Several chemokines and receptors have been shown to be upregulated in the salivary and lacrimal glands of pSS patients potentially leading to migration of T cells to the glands, including the interferon‐induced chemokines CXCL9, CXCL10, CXCL11 and their receptor CXCR3.^4^, ^5^ Recently, CXCR5 + T follicular helper (Tfh) cells and their ligand CXCL13 and CCR9+ ‘Tfh‐like’ cells with their ligand CCL25 have been implicated in disease pathogenesis.^6^, ^7^, ^8^, ^9^, ^10^, ^11^, ^12^, ^13^ Both levels of CXCL13 and CCL25 and numbers of CXCR5 + and CCR9 + Th cells are elevated in the salivary glands of pSS patients. CXCR5 + Tfh and CCR9 + Tfh‐like cells produce pro‐inflammatory cytokines and provide co‐stimulatory signals that strongly stimulate B cell responses which play an important role in B cell hyperactivity pSS.^6^, ^12^, ^13^, ^14^ In addition, these Tfh and Tfh‐like cells may be involved in the formation of germinal centre‐like structures, which are present in the salivary glands of ~25% of pSS patients.^14^ In one study, polarization of circulating Tfh cell subsets towards a Tfh1, Tfh2 or Tfh17 phenotype was not observed in pSS patients, while in another study an increase of Tfh17 was found.^15^, ^16^ In the present study, we investigated co‐expression of chemokine receptors on circulating CCR9 + Th cells, potentially mediating accumulation of effector Th cell subsets in the salivary glands of pSS patients. In addition, we assessed local presence of chemokines in the salivary gland and tested whether increased chemokines may have a concerted action on Th cells.

## 2 PATIENTS AND METHODS

### 2.1 Patients

pSS patients, non‐Sjögren's sicca (non‐SS) patients and healthy subjects were included from the outpatients clinic of the department of Rheumatology & Clinical Immunology of the University Medical Centre Utrecht and were randomly and cross‐sectionally selected. All pSS patients were diagnosed by a rheumatologist and fulfilled the American‐European Consensus Group (AECG) criteria.^17^ The non‐SS patients were defined as patients suffering from ocular and/or oral dryness without any other known cause and who were not diagnosed with an underlying rheumatic disease including Sjögren's syndrome; consequently, they could not be classified using the AECG‐criteria. The study was approved by the hospital's medical ethics committee and all subjects signed informed consent. Patient characteristics are shown in Table 1 for those that contributed to the gene expression, flow cytometry and chemotaxis studies and Table S1 for those that contributed labial biopsies.

**Table 1:** Patients’ characteristics

	Gene expression		Flow cytometry		Chemotaxis	
	non‐SS (n = 9)	pSS (n = 9)	HC (n = 11)	pSS (n = 17)	HC (n = 6)	pSS (n = 10)
Female gender, n (%)	8 (89)	8 (89)	11 (100)	17 (100)	6 (100)	10 (100)
Age, years (Mean ± SD)	53.3 ± 7.5	43.7 ± 19.7	43.4 ± 12.3	54.6 ± 13.2	54.3 ± 6.8	58.6 ± 11.0
Anti‐Ro/SSA positive, n (%)	2 (22)	9 (100)	‐	16 (94)	‐	8 (80)
Anti‐La/SSB positive, n (%)	0 (0)	3 (33)	‐	9 (53)	‐	6 (60)
ANA positive, n (%)	1 (11)	7 (78)	‐	13 (76)	‐	8 (80)
Lymphocytic focus score (foci/4 mm^2^)	0 (0‐0)	3.0 (1.5‐5.0)	‐	2.0 (1.4‐2.7)	‐	2 (1.8‐3)
IgA positive plasma cells (%)	>70	43 (16‐50)	‐	38 (25‐51)	‐	33 (19‐46)
Schirmer (mm/5 min)	1.5 (1.0‐5.0)	4.0 (0.8‐17.0)	‐	3.5 (0.4‐12.3)	‐	4.5 (0.5‐10)
Serum IgG (g/L)	11.4 (11.0‐12.4)	17.4 (10.4‐28.5)	‐	17.2 (13.8‐19.7)	‐	13.6 (12.0‐17.0)
ESR (mm/h)	6 (3‐21)	14 (10‐52)	‐	22 (13‐33)	‐	13 (12‐34)
ESSDAI score (0‐123)	‐	‐	‐	6 (5‐8)	‐	6 (2‐8)
ESSPRI score (0‐10)	‐	‐	‐	6.5 (5‐7)	‐	5 (3‐7)
Immunosuppressants	0	1	‐	3	‐	2
Hydroxychloroquine	‐	0	‐	3	‐	1
Other	‐	1	‐	0	‐	1

Note

Median (IQR, interquartile range) is shown unless specified otherwise.

Abbreviations: ESR, erythrocyte sedimentation rate; ESSDAI, EULAR Sjögren's syndrome disease activity index; ESSPRI, EULAR Sjögren's syndrome patient reported index; HC, healthy controls; non‐SS, non‐Sjögren's sicca; pSS, primary Sjögren's syndrome.

### 2.2 Chemokine and chemokine receptor assessment

Fresh PBMCs from healthy controls (HC) and pSS patients were isolated from heparinized peripheral blood by density centrifugation using Ficoll‐Paque Plus (GE Healthcare). The heparinized blood was left on the bench at room temperature for a maximum of 2 hours. Isolated PBMCs were kept on ice. Freshly isolated PBMCs were stained and analysed by flow cytometry, staining for CD3, CD4, CD45RO, CD27, CCR9, CXCR3, CCR4, CCR6 and CCR10 (Table S2) on the same day. Quantitative PCR was performed using the Applied Biosystem 7000 System (Thermo Fisher Scientific), and TaqMan^®^ Universal PCR Master Mix (Thermo Fisher Scientific). As described,^18^ cDNA was analysed for the expression of CCL17 (Hs00171074_m1), CCL20 (Hs00171125_m1), CCL27 (Hs00171157_m1), CXCL10 (Hs00171042_m1), CCR4 (Hs00747615_s1), CCR6 (Hs00171121_m1), CCR10 (Hs00706455_s1) and CXCR3 (Hs00171041_m1). Expression was normalized to 18S rRNA and consequently was calculated as a fold change relative to the mean of the non‐SS group, using the delta‐delta CT method. All primers were purchased from Thermo Fisher Scientific. Protein levels of these chemokines were measured by Luminex in labial salivary gland biopsy supernatants. Fresh labial salivary gland tissues were thoroughly rinsed and incubated with 200 µL of saline (0.9% NaCl) in a 500 µL vial (Sarstedt) for 1 hour at room temperature. The biopsy tissue was removed from the vial and the remaining tissue supernatants were rendered cell free by centrifugation at 500 *g* for 5 minutes and stored at −80°C. Chemokines were measured using Luminex multiplex technology as previously described.^19^, ^20^

### 2.3 Chemotaxis

Transwell experiments were performed to assess chemotaxis induced by CCL25 and CXCL10. 1 × 10^5^ PBMCs were transferred into the upper chamber of 5 µm pore‐size transwell plates (96 well ChemoTX^®^, NeuroProbe). Fresh medium (RPMI (Gibco) supplemented with penicillin, streptomycin (Gibco) and 10% FBS (Sigma)) alone, or containing CCL25 (0 or 100 ng/mL, Peprotech) and/or CXCL10 (0, 10 or 100 ng/mL, Peprotech) were added to the lower chamber. After 2 hours at 37°C, cells migrating to the lower chamber were quantified by flow cytometry with standardization of the volume and acquisition time per well.

### 2.4 Statistical analysis

Statistical analyses were performed in Prism 6 software and spss. Student's *t* test, paired parametric *t* test, Mann‐Whitney *U* test and Wilcoxon nonparametrical paired test were used where appropriate. For correlations with disease parameters, Pearson's correlation and Spearman's rho were used where appropriate. Differences and correlations were considered statistically significant at *P* < .05, *P* = .05‐.10 was considered as a trend towards statistical significance and indicated in figures.

## 3 RESULTS

### 3.1 Frequencies of circulating memory CXCR3 + CCR9+ Th cells are decreased in pSS

**Figure 1:**
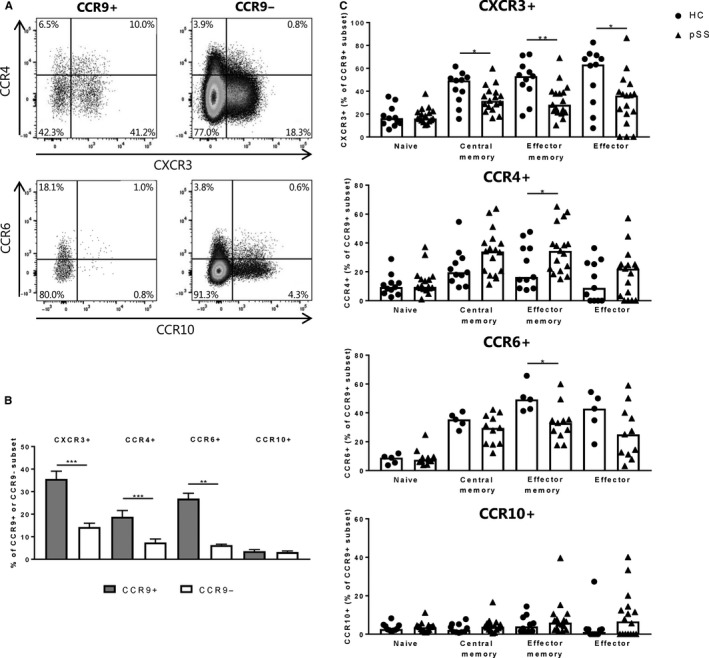
Circulating CCR9 + Th cells have enhanced CXCR3, CCR4 and CCR6 expression and pSS patients show decreased proportions of CCR9 + Th cells expressing CXCR3 and CCR6. Representative flow cytometry images (A) and combined data of multiple healthy donors (n = 5‐11; B) showing increased expression of CXCR3, CCR4 and CCR6, but not CCR10 on circulating CCR9+ vs CCR9− Th cells. Mean with SEM are shown. C, In primary Sjögren's syndrome (pSS) patients circulating CCR9+ central memory, effector memory and effector Th cells expressing CXCR3 and effector memory cells expressing CCR6 are significantly decreased. In addition, circulating CCR9+ effector memory Th cells from pSS patients show elevated percentages of CCR4‐expressing cells. Medians are shown unless specified otherwise, healthy controls (HC): circles, pSS patients: triangles, pSS n = 11‐17, HC n = 5‐11, *P < .05, **P < .01, ***P < .001

Significantly increased frequencies of CXCR3+, CCR4 + and CCR6 + cells were found within the CCR9 + Th cell subset as compared to CCR9‐ Th cells in the circulation of healthy individuals (Figure 1A,B). Interestingly, in pSS patients a significant decrease in CXCR3 + CCR9+ Th cells was found in the central memory (CD45RO + CD27+), effector (CD45RO‐CD27‐) and effector memory (CD45RO + CD27−) subsets, but not naïve Th cells (CD45RO‐CD27+; Figure 1C) in addition, decreased frequencies of CCR6‐expressing CCR9 + effector memory Th cells. In contrast, increased frequencies of CCR4‐expressing CCR9 + effector memory Th cells were observed in pSS patients (Figure 1C). The decreased frequencies of these subsets did not significantly correlate with clinical parameters (data not shown). Trends towards similar differences were observed in CCR9‐ Th cells, although less pronounced (Figure S1). These results indicate a predominant decrease of CXCR3‐expressing CCR9 + memory Th cells in the circulation of pSS patients.

### 3.2 CCL25 and CXCL10 are increased in the salivary glands of pSS patients and synergistically induce Th cell migration in vitro

**Figure 2:**
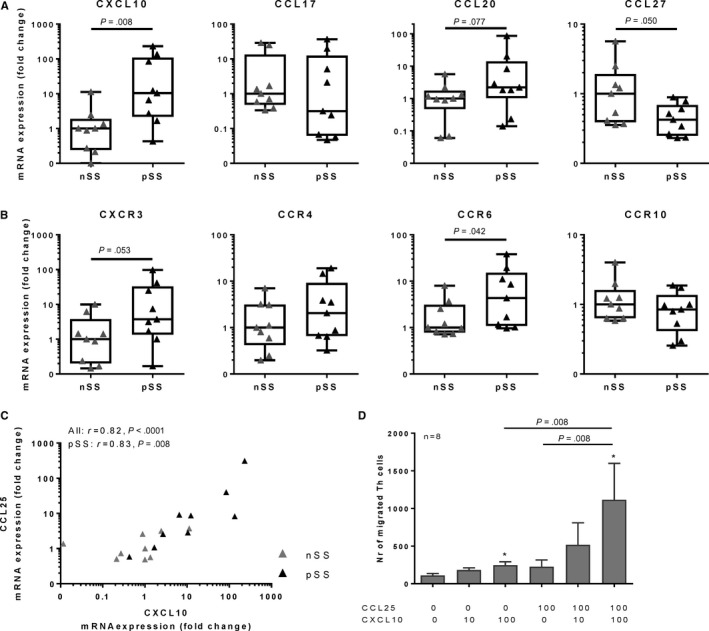
Elevated levels of CCL25 and CXCL10 in salivary glands from pSS patients correlate and in vitro these cytokines induce synergistic chemotaxis of Th cells. A, B, mRNA expression of the following chemokines and their receptors were evaluated in labial salivary gland biopsies (tissue lysates) from primary Sjögren's syndrome (pSS, black triangles) and non‐Sjögren's sicca (non‐SS, indicated as nSS) patients: CXCL10 and CXCR3, CCL17 and CCR4, CCL20 and CCR6 and CCL27 and CCR10, showing elevated expression of CXCL10 and CCR6 and trends towards increases in CXCR3, CCL20 and a decrease of CCL27. Boxplots with medians with interquartile range are shown, non‐SS: n = 9, pSS: n = 9, (C) expression of CXCL10 strongly correlated with CCL25 expression and (D) in a transwell chemotaxis assay, synergistic migration of Th cells is induced upon a combination of CCL25 and CXCL10 (n = 8). Mean with SEM are shown, *P < .05

Next we assessed whether mRNA and protein expression of representative chemokines that can attract CXCR3, CCR4, CCR6 and CCR10‐expressing Th cells were expressed by salivary gland tissue, either expressed as mRNA in tissue lysates or protein levels in supernatant of tissue explants.^21^, ^22^ We previously reported that mRNA expression of CCL25 was significantly increased in salivary glands of pSS patients as compared to non‐SS patients.^12^ Increased CXCL10 mRNA expression as previously reported by others 4 was confirmed (Figure 2A). Also, increased expression of the other CXCR3‐ligands, CXCL9 and CXCL11 in pSS was in line with previous data (fold change (median, interquartile range): 10 (4‐153) and 5 (2‐42) vs non‐SS, *P* = .012 and *P* = .006, respectively).^4^, ^21^ In addition, there was a trend towards increased CCL20 expression (*P* = .077), and a decrease of CCL27 expression (*P* = .050), whereas CCL17 was not differentially expressed (Figure 2A). As we have previously reported, increased protein levels of CCL25 and CXCL10 in secretomes of pSS salivary gland tissue were observed.^4^, ^12^, ^20^, ^22^ We here report that upregulated CCL25 mRNA expression significantly correlated to CXCL10 (Figure 2C). In accordance with the mRNA data these chemokines also correlated on protein level (all sicca donors: *r* = .47, *P* = .002, pSS: *r* = .42, *P* = .04, data not shown in figure). Only very low levels of CCL17 and CCL20 (CCR4 and CCR6 ligands) proteins were found, with increases in only a limited number of patients (data not shown). In addition, CCL27 was present at higher levels, but the levels were not significantly different between healthy controls and pSS patients (data not shown). Increased CCR6 and a trend towards increased CXCR3 mRNA expression were found in the salivary glands of pSS patients (Figure 2B). No significant differences in CCR4 (*P* = .34) and CCR10 (*P* = .29) expression were observed (Figure 2B).

Since a decrease of CXCR3 + CCR9 + Th cells was found in the circulation of pSS patients and both CCL25 and CXCL10 showed the strongest increased expression in the salivary glands on both mRNA and protein level (Figure 2A and 12, 20), the chemotactic capacity of CXCL10 in combination with CCL25 was investigated. In a transwell system, chemotaxis of Th cells in response to different concentrations of CCL25 and CXCL10 was assessed. No differences in migration of Th cells or lymphocytes between HC and pSS was observed, hence the data of these groups were pooled. Interestingly, the combination of CCL25 and CXCL10 synergistically enhanced chemotaxis of CD4 + Th cells as compared to CXCL10 or CCL25 alone (Figure 2D).

## 4 DISCUSSION

In this study, increased co‐expression of chemokine receptors CXCR3, CCR4 and CCR6 on circulating CCR9 + Th cells was found as compared to Th cells lacking CCR9. In the circulation of pSS patients decreased frequencies of CXCR3 + CCR9+ and CCR6 + CCR9 + Th cells were found, which may be due to a concerted action of overexpressed ligands at the site of inflammation. Corroborating this, we found elevated and abundant expression of CXCL10 and CCL25 in the salivary gland and synergistic chemotaxis of Th cells for these cytokines in vitro*.*

This is the first study that shows co‐expression and synergistic function of chemokine receptors on human circulating CCR9 + Th cells. Intestinal CCR9 + Th cells from mice have been found to have elevated expression of CCR6 and CXCR3 and lower expression of CCR10 than CCR9 − Th cells.^23^ Chemokine receptors play an important role in the positioning of antigen‐experienced Th cells in epithelial tissues. CCR4 and CCR10 are regarded as skin‐homing chemokine receptors and CCR9 and CCR6 are involved in intestinal homing.^24^, ^25^ The high co‐expression of CXCR3, CCR4 and CCR6 we found on CCR9 + Th cells as compared to CCR9 − Th cells potentially indicates enhanced homing to the gut but also to other tissues where their ligands are expressed. The chemokine receptor profile corresponds with the pleiotropic cytokine production by this subset, including IFN‐γ, IL‐4 and IL‐17.^12^

In accordance with previous studies by others as well as our group, elevated local expression of the chemokines CCL25 and CXCL10 was found in pSS.^4^, ^12^ Corresponding with the elevated chemokine levels, increased numbers of CCR9 and CXCR3‐expressing cells have previously been found in pSS salivary glands.^4^, ^12^ In this study, we demonstrated a significant correlation of CCL25 and CXCL10 protein expression in labial salivary gland biopsies. A similar correlation was not observed between CCL25 and other chemokines supporting coordinated responses to CCL25 and CXCL10 by CCR9 + Th cells co‐expressing CXCR3. In this study, we demonstrated a significant correlation of CCL25 and CXCL10, which was not observed for CCL25 and the other chemokines on protein level, indicating that effects of CCL25 and CXCL10 on CCR9 + Th cells co‐expressing CXCR3 can coincide. This was corroborated by CCL25 and CXCL10 inducing synergistic chemotaxis of Th cells. In our assay, it was not possible to quantify the number of migrated CCR9‐expressing cells, since CCL25 induces downregulation of CCR9 (Takeda Pharmaceuticals and Blokland et al unpublished data). However, for synergistic chemotaxis of Th cells these cells need to co‐express CCR9 and CXCR3. Hence, we are confident that both cytokines can contribute to enhanced migration of CCR9 + CXCR3 + Th cells.

The important role of CXCR3 in pSS is supported by the recent finding that inhibition of CXCR3 impedes development of pSS‐like disease in a sialadenitis mouse model.^26^ Considering the observation that 40%‐50% of CCR9 + Th cells express CXCR3, it is likely that part of these effects is mediated by blockade of CXCR3 + CCR9 + Th cells. The finding that CCR9 + Th cells have shown to be crucial for experimental Sjögren‐like disease in mice supports this notion.

Although synergistic migration is facilitated by CCL25 and CXCL10, positioning of CCR9 + Th cells is likely regulated by much more complex expression patterns of other chemokines and chemokine receptors and adhesion molecules. This is corroborated by the observation that next to the decreased frequencies of circulating CXCR3‐expressing CCR9 + Th cells in pSS patients frequencies of CCR6‐expressing CCR9 + Th cells were reduced. Although this was only significant for effector memory cells, it should be noted that the number of tested samples was low and addition of more donors may reveal decreased frequencies of CCR6‐expressing cells in the central memory and effector CCR9 + Th cell subsets as well. Despite the fact CCL20 on mRNA and protein level in the salivary gland was not robustly increased, we did observe significantly enhanced CCR6 mRNA in the salivary gland of pSS patients. This suggests that CCL20, which is the only CCR6 ligand, contributes to migration of CCR6‐expressing CCR9 + Th cells. Whether this also induces synergistic migration or sequential or differential spatial migration in the inflamed tissue remains to be studied, but this interaction may play a role in the recruitment of CCR6‐expressing Th17‐polarized CCR9 + Th cells in pSS salivary glands.^27^ In support of a role for CCL20/CCR6, recently it was shown that induction of Sjögren‐like disease is associated by increase in CCL20 concentrations.^28^ Positioning of CCR9 cells is unlikely mediated by CCR10 ligands. A low percentage co‐expresses CCR10, and CCL27 is reduced in the labial salivary gland. The decrease of CCL27 mRNA expression in pSS is in accordance with the decrease of CCL28– the other ligand for CCR10– in saliva of pSS patients.^29^

In this study, we focused on CD4 + Th cells, however, the importance of CD8 + T cells in the immunopathology of pSS has been indicated previously in pSS patients and Sjögren‐like disease models.^30^, ^31^, ^32^, ^33^ In this respect, the interplay between CCR9 + Th cells and (CCR9+) CD8 T cells has been demonstrated previously to play a key role in immunopathology.^13^ Subsets of CD8 T cells have been associated with clinical activity of pSS.^34^ In the current study, we did not study CCR9‐expressing CD8 T cells and CD8 subsets. Future research on chemokine receptor expression and function in CD8 T cells in pSS patients should elucidate the importance of such subsets.

In conclusion, this study indicates that coordinated elevated expression of chemokines in the salivary glands of pSS patients may have synergistic effects on migration of pathogenic CCR9 + Th cells towards the salivary glands. This concept also helps to explain why the CXCR3 + CCR9 + Th subset may be reduced in the circulation of pSS patients.

## CONFLICT OF INTEREST

None.

## AUTHOR CONTRIBUTIONS

All authors were involved in drafting the article or revising it critically for important intellectual content, and all authors approved the final version to be published. Dr Van Roon had full access to all of the data in the study and takes responsibility for the integrity of the data and the accuracy of the data analysis. Study conception and design: Blokland, Kislat, Homey, Smithson, Kruize, Radstake, Van Roon. Acquisition of data: Blokland, Kislat, Kruize. Analysis and interpretation of data: Blokland, Kislat, Homey, Smithson, Kruize, Radstake, Van Roon.

## DISCLOSURES

This study was funded by Takeda Pharmaceuticals. GM Smithson is an employee of Takeda Pharmaceuticals.
