# Supplementary material for: Decreased circulating CXCR3 + CCR9+T helper cells are associated with elevated levels of their ligands CXCL10 and CCL25 in the salivary gland of patients with Sjögren’s syndrome to facilitate their concerted migration
Source: Scand J Immunol. 2019 Dec 13;91(3):e12852. doi: 10.1111/sji.12852 (PMC7064901; doi:10.1111/sji.12852)
Supplement: Supplementary file 2 [file SJI-91-e12852-s002.docx]

**Supplementary tables**

|  | **non-SS**  **(n=17)** | **pSS**  **(n=24)** |
| --- | --- | --- |
| **Female gender, n (%)** | 15 (88) | 21 (88) |
| **Age, years (mean ± SD)** | 49 ± 16 | 55 ± 12 |
| **Anti-Ro/SSA+, n (%)** | 0 (0) | 16 (67) |
| **Anti-La/SSB+, n (%)** | 0 (0) | 4 (17) |
| **ANA+, n (%)** | 9 (53) | 17 (71) |
| **Lymphocytic focus score (foci/4mm^2^)** | 0  (0 – 0) | 1.6  (1.0 – 2.6) |
| **IgA positive plasma cells (%)** | 77  (71 – 80) | 59  (49 – 64) |
| **Schirmer (mm/5min)** | 4  (1 – 7) | 7  (3 – 11) |
| **Serum IgG (g/L)** | 11.0  (6.5 – 12.3) | 13.9  (10.1 – 18.3) |
| **ESR (mm/h)** | 7  (6 – 10) | 15  (7 – 29) |
| **ESSDAI score (0-123)** | NA | 3  (1 – 7) |
| **ESSPRI score (0-10)** | NA | 5.3  (2.3 – 6.0) |
| **Immunosuppressants, n (%)** | 1 (6) | 3 (12.5) |

***Supplementary table 1. Patients’ characteristics salivary gland biopsy supernatants.*** *Median (IQR, interquartile range) are shown unless specified otherwise. non-SS: non-Sjögren’s sicca; pSS: primary Sjögren’s syndrome; HC: healthy controls; ESR: erythrocyte sedimentation rate; ESSDAI: EULAR Sjögren’s syndrome disease activity index; ESSPRI: EULAR Sjögren’s syndrome patient reported index.*

| Marker | Fluorochrome | Company | Clone |
| --- | --- | --- | --- |
| CD3 | AF700 | Sony Biotechnology | UCHT1 |
| CD4 | PerCP-Cy5.5 | Sony Biotechnology | RPA-T4 |
| CD45RO | BV711 | Biolegend | UCHL1 |
| CD27 | APC-ef780 | eBioscience | O232 |
| CCR9 | PE | Bio-Techne/R&D | 248621 |
| CXCR3 | FITC | Biolegend | G025H7 |
| CCR4 | BV605 | BD | 1G1 |
| CCR6 | PE-Cy7 | eBioscience | R6H1 |
| CCR10 | APC | R&D systems | 314305 |

***Supplementary table 2.*** *Antibodies used for flow cytometry.*
