# Supplementary material for: Decreased circulating CXCR3 + CCR9+T helper cells are associated with elevated levels of their ligands CXCL10 and CCL25 in the salivary gland of patients with Sjögren’s syndrome to facilitate their concerted migration
Source: Scand J Immunol. 2019 Dec 13;91(3):e12852. doi: 10.1111/sji.12852 (PMC7064901; doi:10.1111/sji.12852)
Supplement: Supplementary file 3 [file SJI-91-e12852-s003.docx]

**Supplementary figure 1. Chemokine receptor expression analysis on circulating CCR9- Th cells in HC versus pSS.** Similar to CCR9+ Th cells, trends towards decreased percentages of CXCR3 and CCR6 expressing cells are found in circulating CCR9- Th cells from pSS patients. . Medians are shown, healthy controls (HC): circles, pSS patients: triangles, pSS n=11-17, HC n=5-11, *: p<0.05, **: p<0.01, ***:p<0.001.
